# 25-hydroxycholesterol: an integrator of antiviral ability and signaling

**DOI:** 10.3389/fimmu.2023.1268104

**Published:** 2023-09-13

**Authors:** Jialu Zhang, Yaohong Zhu, Xiaojia Wang, Jiufeng Wang

**Affiliations:** ^1^ College of Veterinary Medicine, China Agricultural University, Beijing, China; ^2^ College of Veterinary Medicine, Sanya Institute of China Agricultural University, Sanya, China

**Keywords:** oxysterol, antiviral effect, cholesterol metabolism, signaling regulation, interferon-independent pathways

## Abstract

Cholesterol, as an important component in mammalian cells, is efficient for viral entry, replication, and assembly. Oxysterols especially hydroxylated cholesterols are recognized as novel regulators of the innate immune response. The antiviral ability of 25HC (25-Hydroxycholesterol) is uncovered due to its role as a metabolic product of the interferon-stimulated gene CH25H (cholesterol-25-hydroxylase). With the advancement of research, the biological functions of 25HC and its structural functions have been interpreted gradually. Furthermore, the underlying mechanisms of antiviral effect of 25HC are not only limited to interferon regulation. Taken up by the special biosynthetic ways and structure, 25HC contributes to modulate not only the cholesterol metabolism but also autophagy and inflammation by regulating signaling pathways. The outcome of modulation by 25HC seems to be largely dependent on the cell types, viruses and context of cell microenvironments. In this paper, we review the recent proceedings on the regulatory effect of 25HC on interferon-independent signaling pathways related to its antiviral capacity and its putative underlying mechanisms.

## 1 Introduction

Cholesterol has occupied the attention of scientists and clinicians for decades because of its physiological and pathological importance in different diseases. As the most abundant lipids in mammalian cells, it predominantly localizes to plasma membranes and regulates the rigidity, fluidity and permeability of lipid bilayer by adjusting the cholesterol concentrations and interacting with adjacent lipids such as sphingolipids (1–3). The interaction of cholesterol and related proteins forms a special structure to modulate the transmission among membranes and signal transduction. Moreover, the distribution of cholesterol in different membranes affects the process of viral infection including its entry, replication and release (4, 5). In the past decade, scientists had focused on the role of cholesterol in host immunity to pathogens. Multiple pieces of evidence proved that some kinds of viruses need cholesterol to enter organisms, moreover, the homeostasis of cholesterol metabolism was disturbed during the invasion of viruses (6, 7). Targeting cholesterol became a novel antiviral strategy (8, 9).

Oxysterol is one of the downstream metabolites of cholesterol, synthesized via enzyme-dependent or enzyme-independent oxidative reaction, which possesses an additional hydroxyl, epoxide or ketone in the sterol nucleus and/or a hydroxyl group in the side chain in the structure (10). In the past, the investigation of oxysterols was mainly focused on the physiological roles in the synthesis of sterol derivates, sterol metabolism and gene regulation (11). Recently, the beneficial effect of some side-chain oxysterols like 25-hydroxycholesterol (25HC) raises lots of attention and gets proven, such as its antibacterial and antiviral properties (12, 13). 25HC was shown to have the ability to modulate cholesterol metabolism thus regulating cholesterol biosynthesis (14). Its ability related to immunity was hinted by the discovery of the enzyme CH25H (cholesterol-25-hydroxylase), which catalyzes the synthesis of 25HC and was upregulated in immunocytes exposed to inflammatory agents (15–17). CH25H was proved as a member of the interferon-stimulating genes (ISGs) family (18), meanwhile, 25HC seems to have similar features to CH25H and is augmented in macrophages after viral infection and by interferon signaling and shows great potential to counteract the enveloped viruses (19). Although the antiviral abilities of 25HC were demonstrated by pieces of investigations, however, whether 25HC exerts antiviral capacity via interferon-independent signaling pathways is yet to be well elucidated. Herein, we review the regulation of 25HC biosynthesis and cholesterol metabolism and discuss the interferon-independent pathways 25HC utilized, especially autophagy, inflammation, TGFβ and posttranslational modification, to exert immunomodulating and antiviral effects.

## 2 Viral infection and host cholesterol

Viruses are proven to regulate host metabolism for their pathogenic effects. With regard to enveloped viruses, cholesterol inevitably participates in the viral infection because of the infective processes including the fusion of the virus-cell membrane and virus-endosome membrane and the endocytosis of viruses (20). The viral infection directly results in the rearrangement of cellular plasma membrane, the formation of host cubic membranes is induced by both enveloped and non-enveloped viruses (21, 22). The cubic membranes provide a special place for viral genome replication and particle assembly and protect the viruses from the recognition of the host immune system (23). The formation of cubic membranes is notably relevant to the expression of HMG-CoA reductase and the negative feedback caused by the administration of cholesterol (24).

Interferon was shown to benefit the antiviral process of host, in the last years, a sort of transmembrane protein called interferon-induced transmembrane (IFITM) protein was identified as restriction factor for SARS-CoV (Severe Acute Respiratory Syndrome Coronavirus), ZIKV (Zika Virus), HIV-1 and IAV (Influenza A Virus) (25, 26). IFITM3 restricts viral entry by interacting with Vesicle-membrane-protein-associated protein A and thus disrupts the host cholesterol homeostasis (27), meanwhile, the formation of fusion pore is blocked at the virus-endosome hemifusion by IFITM3, which also induces accumulation of cholesterol in late endosome/lysosome (28, 29). Of note, the amphipathic helix of IFITM3 was found to alter lipid membrane and directly interact with cholesterol, which plays vital roles in antiviral function and may lead to the blockage of fusion pore formation (26). Stimulator of interferon genes (STING), a transmembrane protein located in the endoplasmic reticulum (ER), is vital for type I interferon response (30). The translocation of STING from ER to Golgi is essential for the activation of downstream protein and host immunity (31). The decreased cholesterol flux can induce type I interferon response in a STING-dependent manner, which can be eliminated by adding free cholesterol (32), moreover, a recent study clarifies that cholesterol in the Golgi membrane is essential for the activation of STING (33).

The lipid rafts are cholesterol-rich lipid domains on the host plasma membrane. The enhanced cholesterol levels benefit the lipid rafts formation and therefore benefit the viral entry or adsorption (34, 35). The depletion of cholesterol in membranes by methyl-β-cyclodextrin effectively suppresses the infectivity of Porcine Reproductive and Respiratory Syndrome Virus and SARS-CoV (6, 36). Moreover, the host cholesterol level and cholesterol metabolism are essential for viral entry, results of CRISPR screening showed that cholesterol metabolism is a crucial pathway for coronavirus infection, viruses target and reprogram cholesterol metabolism for enhanced cholesterol and facilitated viral replication (37). Based on the antiviral strategy targeting cholesterol, 25-hydroxycholesterol as part of the cholesterol-negative feedback loop comes into notice and displays antiviral potential.

## 3 25HC biosynthesis

**Figure 1:**
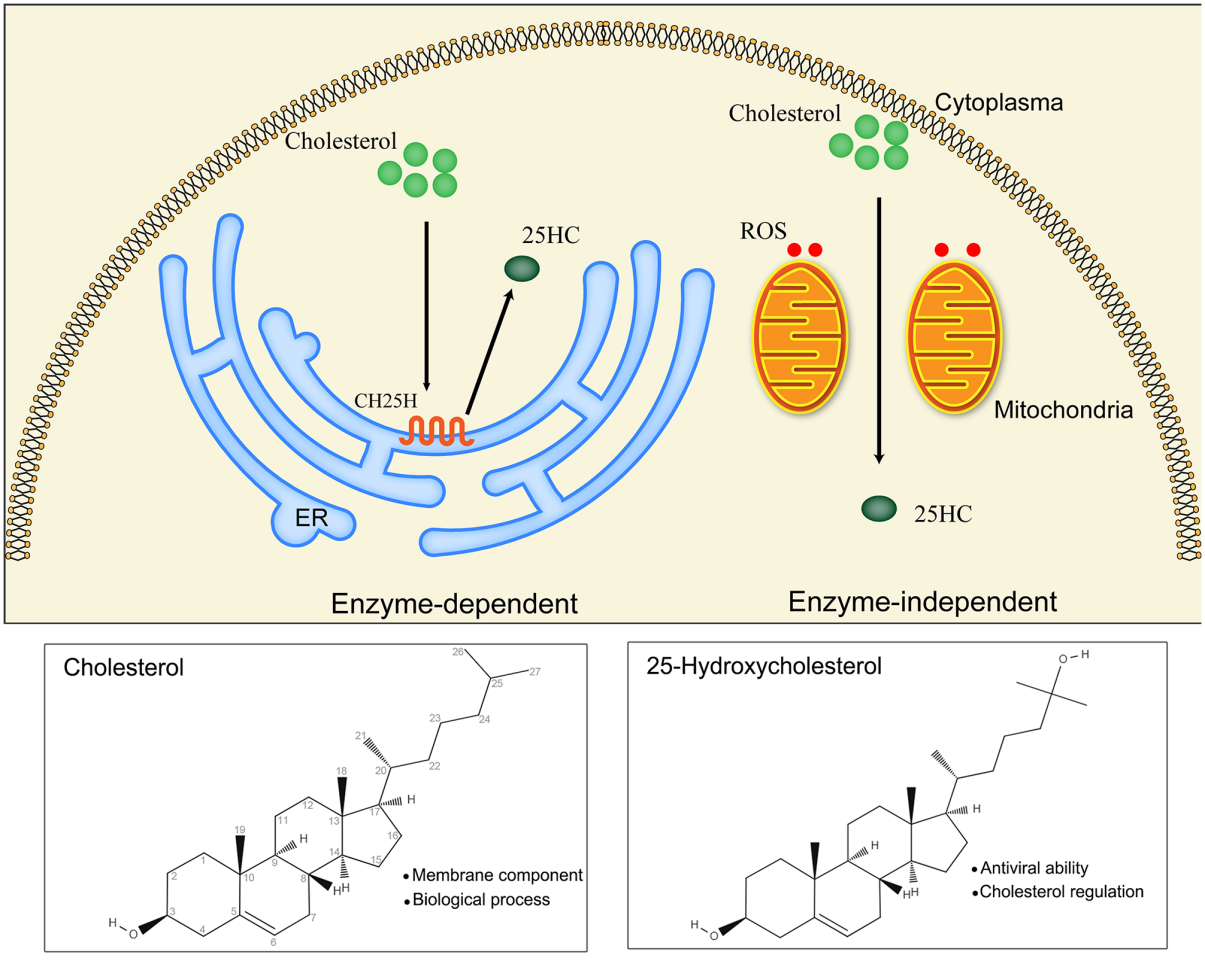
Main biosynthetic approaches of 25HC (25-Hydroxycholesterol): enzyme-dependent and enzyme-independent. The enzymatic reaction catalyzed by CH25H (cholesterol 25-hydroxylase) is the main approach for 25HC production. CH25H is a multi-transmembrane protein located in the endoplasmic reticulum (ER). The non-enzymatic reaction is mediated by ROS (reactive oxygen species) in the existence of mitochondria.

25HC is produced from cholesterol by the hydroxylation reaction occurring at position 25, which results in a more hydrophilic structure than cholesterol (38). This hydroxylation reaction of 25HC is catalyzed by CH25H, which belongs to the oxidoreductase family and mainly localizes in the ER (39). In addition, CH25H is an interferon-stimulating gene showing modulating effects on the immune system through intrinsic and extrinsic pathways and is proven to directly restrict the entry of viruses (40–42). Besides, there is another approach capable of generating 25HC *in vitro* except CH25H. The formation of 25HC was observed when cholesterol was incubated with rat liver mitochondria, NADPH and oxygen, besides, the conversion of cholesterol to 25HC was detected in the incubation solution comprised of Tris-HCl buffer (containing isocitrate and glycerol) and porcine liver mitochondria (43), however, 25HC could not generate by cholesterol and mitochondria without oxygen and NADPH. Evidence indicated that 25HC can be produced by ROS in a non-enzymatic manner (44, 45) (**Figure 1**). Although the 25HC is important for cholesterol homeostasis, its levels in blood and tissues under normal condition are very low, and the expression of CH25H in tissues is low as well (46). The 25HC was almost undetectable in the human plasma after the autooxidation correction of cholesterol (47), which implies the potential of 25HC to be monitored in the progress of diseases. However, the determinants of the approach for 25HC synthesis needs to be further demonstrated.

## 4 25HC and cholesterol metabolism

In the last years, cholesterol level is shown to affect viral infection and host immunity, regulation of genes related to cholesterol biosynthesis, homeostasis and esterification play important roles in resisting viruses.

### 4.1 SREBPs

As a metabolite of cholesterol metabolism, oxysterols exert important roles in the negative feedback loop of sterol biosynthesis (48). Sterol response element-binding proteins (SREBPs), a sort of transcription factor derive from ER, are responsible for cellular cholesterol biosynthesis (in particular SREBP2) (49). In the case of absent cholesterols, SREBP2 is synthesized from ER and then binds to SREBP cleavage-activating protein (SCAP), after that, the SREBP-SCAP complex translocate to the Golgi to become active state processed by proteases site 1 (serine protease) and site 2 (metalloprotease) (50). The active SREBPs promote cholesterol biosynthesis by inducing related enzymes, such as HMGCR (HMG-CoA reductase) (51). When the content of cholesterol is excessive, the structure of SCAP is modified and SCAP-INSIG (insulin-induced gene) complex is formed, which retains SREBPs in the ER, causing disturbance of the SREBPs process and inhibition of cholesterol biosynthesis (52). 25HC functions by binding to INSIG and modifying its structure instead of modifying the conformation of SCAP (53).

**Figure 2:**
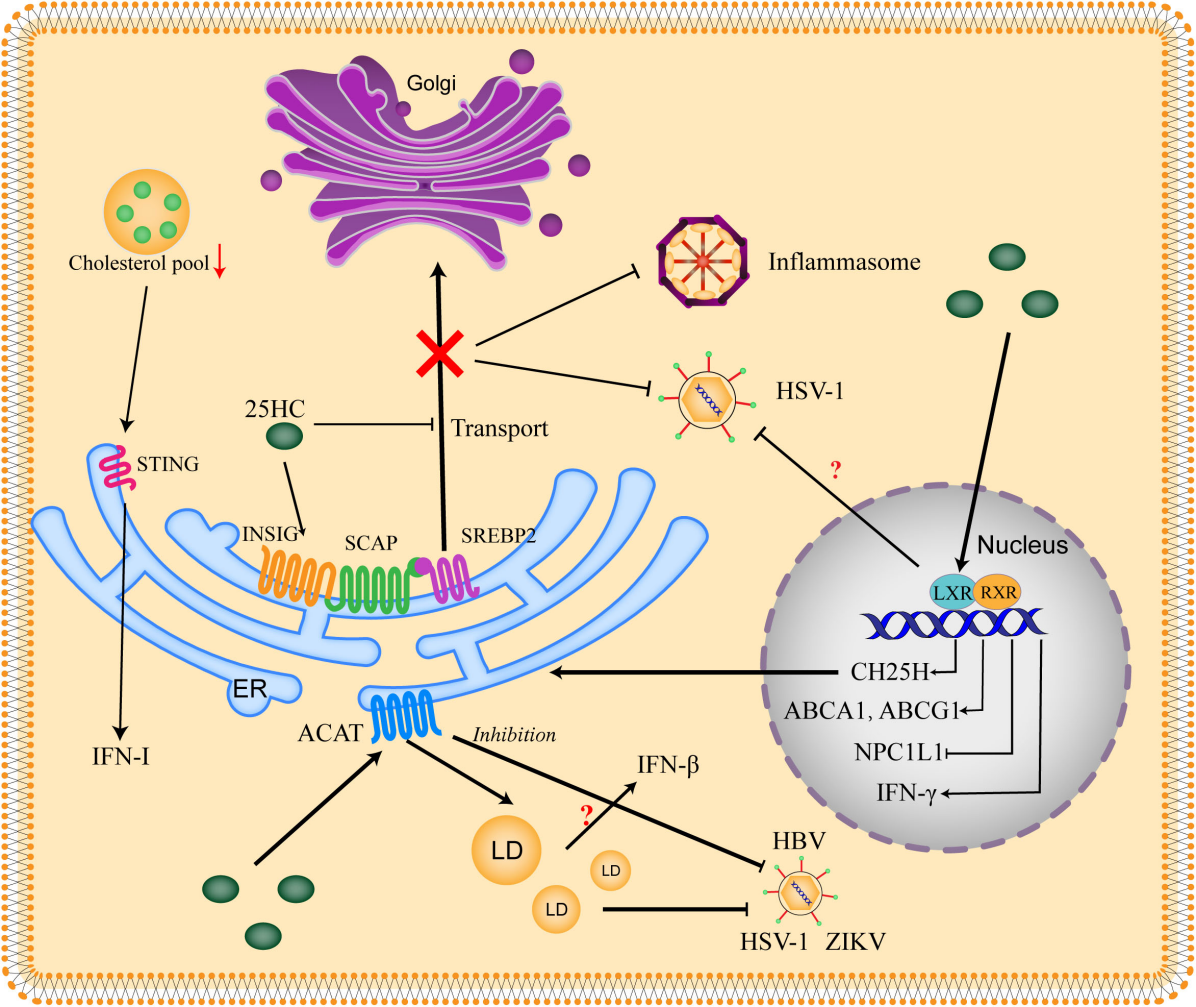
Relationship between 25HC and cholesterol metabolism in antiviral host defense. The SREBP2 (sterol regulatory element-binding protein 2) is critical for cholesterol biosynthesis. SREBP2 is synthesized on the ER and interacts with SCAP (SREBP-cleavage activating protein), then the SREBP2 is translocated to the Golgi for activation. When the cholesterol is excess, the INSIG (insulin-induced gene) is recruited by SCAP and the SCAP-SREBP2-INSIG complex is formed to retain the SREBP2 in the ER. 25HC blocks the transport of SREBP2 through binding to INSIG and promoting the complex formation, thus suppressing the production of cholesterol to impede viral infection. Excess cholesterol is esterized by ACATs (acetyl-CoA acetyltransferases) to store in LDs (lipid droplets). 25HC restricts viral replication by promoting cholesterol esterification and LDs formation, which might be related to IFN-β production. The inhibition of ACAT also shows an antiviral effect. 25HC serves as a ligand of LXR (Liver X receptor) to form LXR/RXR (Retinoid X receptor) complex, which binds to the promoter of LXR response elements and thus stimulates transcription of targets genes including CH25H, ABCA1 (ATP-binding cassette subfamily A member 1), ABCG1 (ATP-binding cassette subfamily G member 1) and NPC1L1 (Niemann-Pick type C1-like 1). Moreover, IFN-γ can be induced by LXR. 25HC is shown to inhibit virus in an LXR-dependent or LXR-independent manner, the context dependence and virus dependence of LXR need to be further explored. The decrease of cholesterol pool induces IFN-I (type-I interferon) response in a STING (Stimulator of interferon genes)-dependent manner.

The antiviral activity of 25HC has been broadly proved by multiple studies, of note, the paracrine and autocrine antiviral response provided by 25HC against viral infection is consistent with the inhibition of cholesterol synthesis by restricting the processing of SREBP2. The researchers find that 25HC exert cellular antiviral effects through SREBP-dependent and LXR (Liver X Receptor)-independent pattern (16), interestingly, the antiviral capacity of 25HC is markedly promoted when the lipid is consumed, in which the SREBP processing is activated (19). Moreover, oxysterols targeting the SREBP pathway, such as 27HC and 24(S), 25-epoxycholesterol, can inhibit viral infection under lipid-consumed conditions (19). Besides, pieces of evidence show that antagonism of SREBPs can impede West Nile virus (WNV), hepatitis C virus (HCV) and Andes virus infections (54–56). The relationship between 25HC and cholesterol metabolism in antiviral host defense has been shown in **Figure 2**.

SREBPs have also been shown to regulate inflammation directly. SREBP2 is activated by atheroprone flow in endothelial cells and then induces expression of NLRP3 (NOD-like Receptor Protein 3), thus enhancing endothelial inflammation (57). In the CH25H-deficient macrophages, 25HC can’t be produced but interleukin-1 (IL-1) family cytokines are spawned, furthermore, 25HC can disrupt the processing of SREBP to restrain the production of IL-1 and aggravation of inflammation (58). It seems that SREBPs exert various effects upon inflammasomes and might play important roles in regulating virus-induced inflammation of organisms, which needs to be uncovered in the future.

### 4.2 LXR

LXRs, a kind of transcription factor sensitive to sterols, play diverse roles in biological processes, especially in lipid regulation (59). Oxysterols including 25HC are proven as ligands of LXRs (LXRα and LXRβ), which can activate the expression of LXRs under the condition of high cholesterol (60). The targets of LXR contain genes related to cholesterol metabolism, such as CH25H, SULT2B1b (cholesterol sulfotransferase-2B1b), and genes related to cholesterol effluxes, such as ABCA1 (ATP-binding cassette subfamily A member 1), ABCG1 (ABC subfamily G member 1), ABCG5 and ABCG8, which can be upregulated by LXRs to enhance the cholesterol efflux (61, 62). *GRAMD1* genes encoding Asters proteins belong to a family of sterol transporter, which is important to sense the level of accessible cholesterol and form the membrane contact sites (63), evidence has shown that the Gramd1b gene can be a transcriptional target of LXRs (64). Moreover, the activated LXRs can negatively regulate the expression of NPC1L1 (65), of which the underlying mechanisms are yet to be elucidated.

The activation of LXR can inhibit infection of HSV-1 (herpes simplex virus type 1) or VSV in a HepG2 cell model, and the inhibition of LXR makes the cell more susceptive to VSV and attenuates the antiviral effect of 25HC (66). The specific oxysterols and relevant enzyme are important for LXR functions in various cell types such as immunocytes (67). 25HC induces the expression of CH25H by upregulating LXR (68), meanwhile, 25HC also enhances IFN-β expression in a LXR-dependent manner. As a result, an antiviral model of 25HC is uncovered, in which the activation of LXR plays an important role (66). However, as we mentioned above, 25HC exerts antiviral effects against multiple viruses in a LXR-independent manner (19). The cell type dependence, disease dependence, virus dependence and LXR isoform dependence might be the critical factors affecting the mechanisms, which remains unclear.

In summary, these observations display that the ability of 25HC to restrain the synthesis of cholesterol and its role as a ligand for nuclear receptors make it exert antiviral effects.

### 4.3 Cholesterol esterification and lipid droplets

The esterification of cholesterol is an important part of cholesterol metabolism, which can mitigate the toxicity of accumulated free cholesterol in cells, mediated by ACATs (acetyl-CoA acetyltransferases), and manages the storage in lipid droplets (LDs) (69). There are two isozymes of ACAT in mammals, ACAT1 and ACAT2 (70). ACAT1 distributes in the whole body and mainly in macrophages and epithelial cells (71). ACAT2 is abundantly expressed in enterocytes and hepatocytes (72).

The application of ACAT inhibition or knockdown is shown to suppress the growth of tumors, which extends to the therapy of cancerogenic viruses, such as HBV (73, 74). The application of ACAT inhibitor, K604, induces CD8+ T cells specific to HBV in the peripheral blood mononuclear cells and boosts CD4+ T cells in the liver. The addition of ACAT inhibitor shows no effect on cccDNA (the original template of HBV viral RNA) or HBeAg in the human HepG2-NTCP (sodium-taurocholate cotransporter polypeptide) cells model. However, the addition significantly reduces the extracellular HBV DNA and HBsAg. The initiating addition of the inhibitor confirms its antiviral ability to the genesis of HBV particles (75). Furthermore, the apolipoproteins are shown to regulate HBV and increase the infectivity of HBV particles (76), which further indicates the ability to inhibit ACAT to retard HCV particles (77). A selective inhibitor of ACAT, SZ58-035, is administrated in a SARS-CoV-2 pseudovirus model, the results clarify that the inhibition of ACAT can suppress the viral entry. The knockdown of ACAT using shRNA leads to promoted viral entry in the presence of 25HC. In addition, the ability of 25HC to promote ACAT activity is confirmed by staining the dynamic of lipid droplets in cells (78). In a Calu-3 cell model infected by SARS-CoV-2, 25HC restricts the viral infection by activating the ACAT, which results in the translocation of accessible cholesterol in the plasma membrane (79) (**Figure 2**).

Apart from the antiviral ability of ACAT per se, the storage organelle of its enzymatic product has obtained greater attention. LD is a kind of organelle containing neutral lipids such as triacylglycerols and cholesterol esters, which originated from ER and regulated by enzymes diacylglycerol acyltransferases (DGATs) and ACATs (69). In the investigation of cancer, the opposing effects of lipid droplets are shown in tumor cells and immune cells, stimulating cancer cell growth and invasion and impairing immune cell function respectively (80–82). The dual effects of lipid droplets are also discovered in viral infection. Early studies have shown that LDs participate in the life cycle of multiple RNA viruses, such as HCV and DENV. The viral capsid proteins anchor on LDs to form key sites for particle production (83, 84). In enteroviruses infection, the novel mechanism of LDs is uncovered to create replication compartments by recruiting host LDs, moreover, the viral proteins interact with the lipolysis pathway to utilize fatty acids to facilitate its replication. Whereas the inhibition of recruiting LDs and the contact between LDs and replication compartments impedes enterovirus replication (85). Recent studies have focused on the antiviral effect of LDs. During the early infection of HSV-1, IAV, DENV and ZIKV *in vitro*, LDs are predominantly upregulated, the LDs in lung sections of C57BL/6 mice infected by IAV are also increased when compared with the mock mice. Furthermore, the induction of LDs by oleic acid is accompanied by dramatically increased IFN mRNA and protein levels. In the presence of induced LDs by oleic acid treatment in astrocytes, the infection of ZIKV is followed by the increased production of INF-β mRNA and the reduced viral load is correlated with the upregulated IFN-β and LDs (86). The ability of 25HC to stimulate the formation of lipid droplets is confirmed in various studies (13, 79). The antiviral effect of 25HC on SARS-CoV-2 is consistent with the induction of LDs (79). In our previous study, the early infection of PDCoV (porcine deltacoronavirus) at 8 h to some extent induces the accumulation of LDs, 25HC treatment induces abundant LDs and reduces the replication of PDCoV accompanied by upregulated IFN-β mRNA (87). The opposite effects of LDs demonstrate its antiviral potential by enhancing the interferon response to viral infection, meanwhile, indicate the inverse mechanisms utilized by viruses to hinder the early antiviral response of organisms.

Taken together, these findings above indicate that LDs production mediated by ACAT plays critical roles in antiviral response, which might be a novel target of 25HC.

## 5 25HC and autophagy

**Figure 3:**
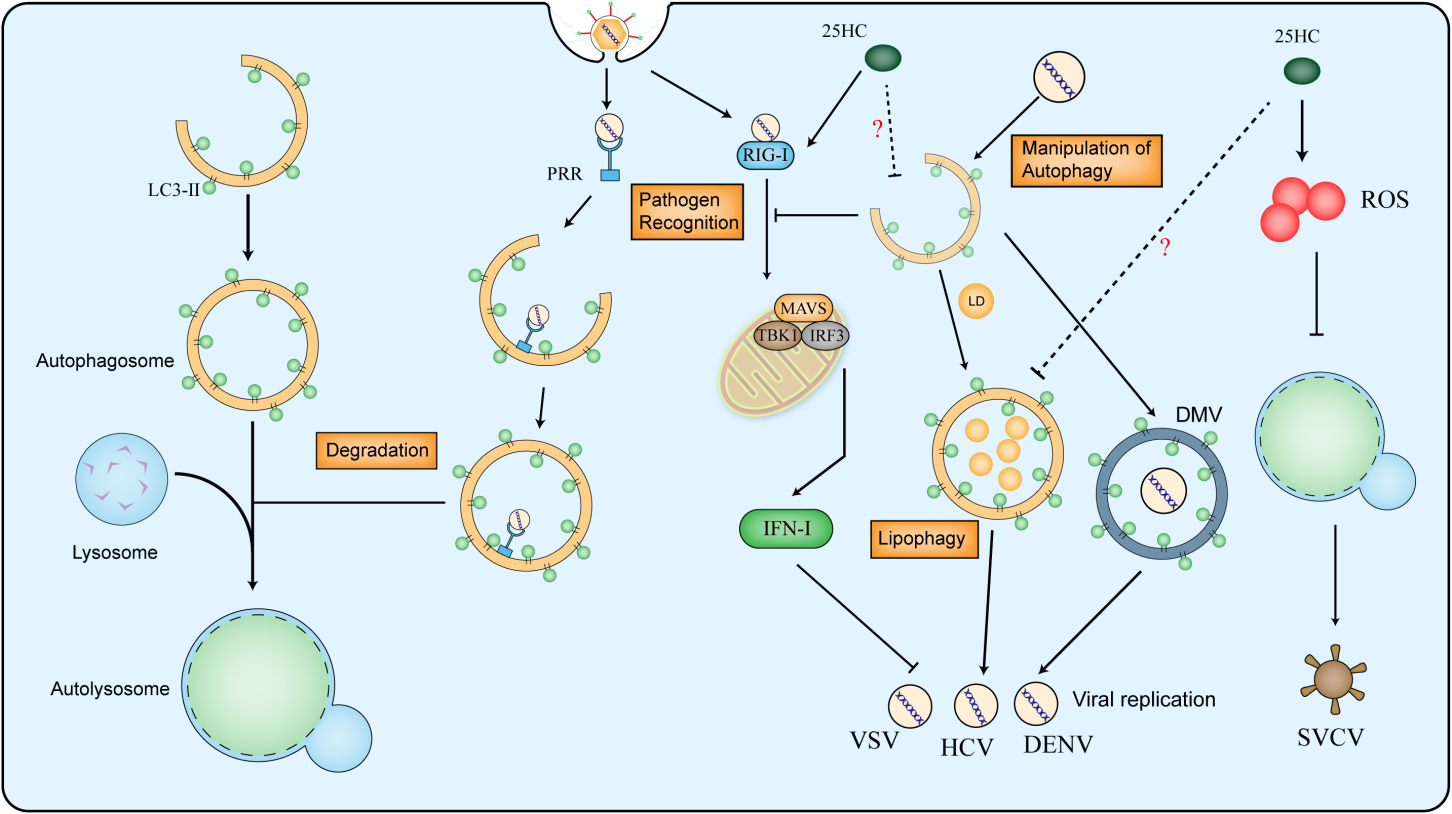
Relationship between virus and autophagy and the effect of 25HC on autophagy in antiviral responses. The whole process of autophagy contains the formation of autophagosome, fusion of lysosome and formation of autolysosome. The LC3-II is used as marker of autophagosome in mammalian cells. The PRR (pattern recognition receptors) can recognize the viral molecules and activate autophagy for viral degradation, However, the RIG-I (retinoic acid-inducible gene I) senses viral double-stranded RNA to activate MAVS (Mitochondrial Antiviral-signaling protein), IRF3 (Interferon Regulatory Factor 3) and TBK1 (Tank-binding Kinase 1, the downstream of SING protein) and promote type I IFN production, which can be suppressed by autophagy. Viruses can utilize autophagy to form DMV (double-membrane vesicle) and to provide energy for its replication. 25HC restricts virus infection by inhibiting autophagy in a ROS-mediated way, moreover, 25HC induces expression of RIG-I and downstream genes, the potential mechanism among 25HC, RIG-I and autophagy in the antiviral response still needs to uncover.

Autophagy is a conserved cellular process that is involved in the degradation of proteins, lipids and organelles, to maintain the homeostasis of organisms. The content in vehicles that needs to be degraded is delivered to lysosomes through three primary approaches: macroautophagy, microautophagy and chaperone-mediated autophagy (CMA) (88). As a double-edged sword, autophagy is considered as part of the immune system to eliminate microorganisms, whereas pathogens evolve reciprocal mechanisms to fight back. Autophagy can be induced by PRRs (pattern recognition receptors) recognizing pathogen-associated molecules. In the early stage of infection, stimulated Toll-like receptor (TLR) recruits MYD88 (adaptor myeloid differentiation primary response protein) to bind to Beclin1 and hence upregulates the level of autophagy (89). The RLRs (RIG-I-like receptors) sense viral nucleic acid to activate downstream of the interferon pathway and the production of IFN-I, nevertheless, autophagy is shown to suppress the immune response triggered by RLRs (90, 91). Moreover, some specific proteins interact with viral proteins and lead to autophagosomes for degradation, which ultimately inhibits viral proliferation (92, 93). Some viruses evolve to utilize the double-membrane structure of autophagosome to protect viral RNAs from recognition by the immune system and provide shelter for its replication (94, 95). Pieces of evidence have proved the ability of coronavirus to utilize autophagy (96–98), in which selective autophagy also plays critical roles, such as mitophagy and lipophagy (99, 100). The formation of double-membrane vesicle (DMV) shelters the viral RNA from recognition and degradation, meanwhile, the lipophagy provides energy for viral replication (**Figure 3**).

Some oxysterols are shown to activate autophagy in the studies of atherosclerosis, resulting in the increase of autophagosomes and LC3-II (101). 7-Ketocholesterol (7-KC) induces LC3-II/LC3-I expression and autophagosome formation in the smooth muscle cells (102). Moreover, 7-KC is shown to trigger autophagy in an enzyme-dependent manner by promoting the level of cellular ROS (103). Nevertheless, 25HC seems to play inverse roles in regulating autophagy. In a study of non-small cell lung cancer, 25HC treatment possesses the ability to inhibit autophagy in the presence of BIX (104). In addition, treatment with 25HC induces the expression of RIG-I and downstream genes (105), which implies the potential regulatory effect of 25HC on autophagy. The effect of 25HC on autophagy is verified by simultaneously administrating with autophagy modulators after SVCV (spring viremia of carp rhabdovirus) infection, the combination of 25HC and autophagy inhibitor increased the SVCV neutralization, whereas the autophagy activator increases the infectivity of SVCV. Furthermore, the addition of an oxidative stress inhibitor indicates that 25HC might block the autophagy process by inducing the accumulation of ROS (106).

It seems that oxysterols might modulate the outcome of autophagy in different manners, but its concentration dependence, cell type and disease dependence remain uncertain. On the other hand, the combat of autophagy and viruses is a complex process, to what extent and under what condition the 25HC exerts antiviral effects by interfering with autophagy is yet to be elucidated.

## 6 25HC and inflammation

Persistent viral infection always induces inflammation activation, proper extent of inflammation benefits the clearance of pathogens and the promotion of host immunity, whereas excess inflammation brings a burden to the organism and aggravates the illness (107). Therefore, ideal therapeutic medicines against viruses should also possess the ability to modulate inflammation for immunity homeostasis.

**Figure 4:**
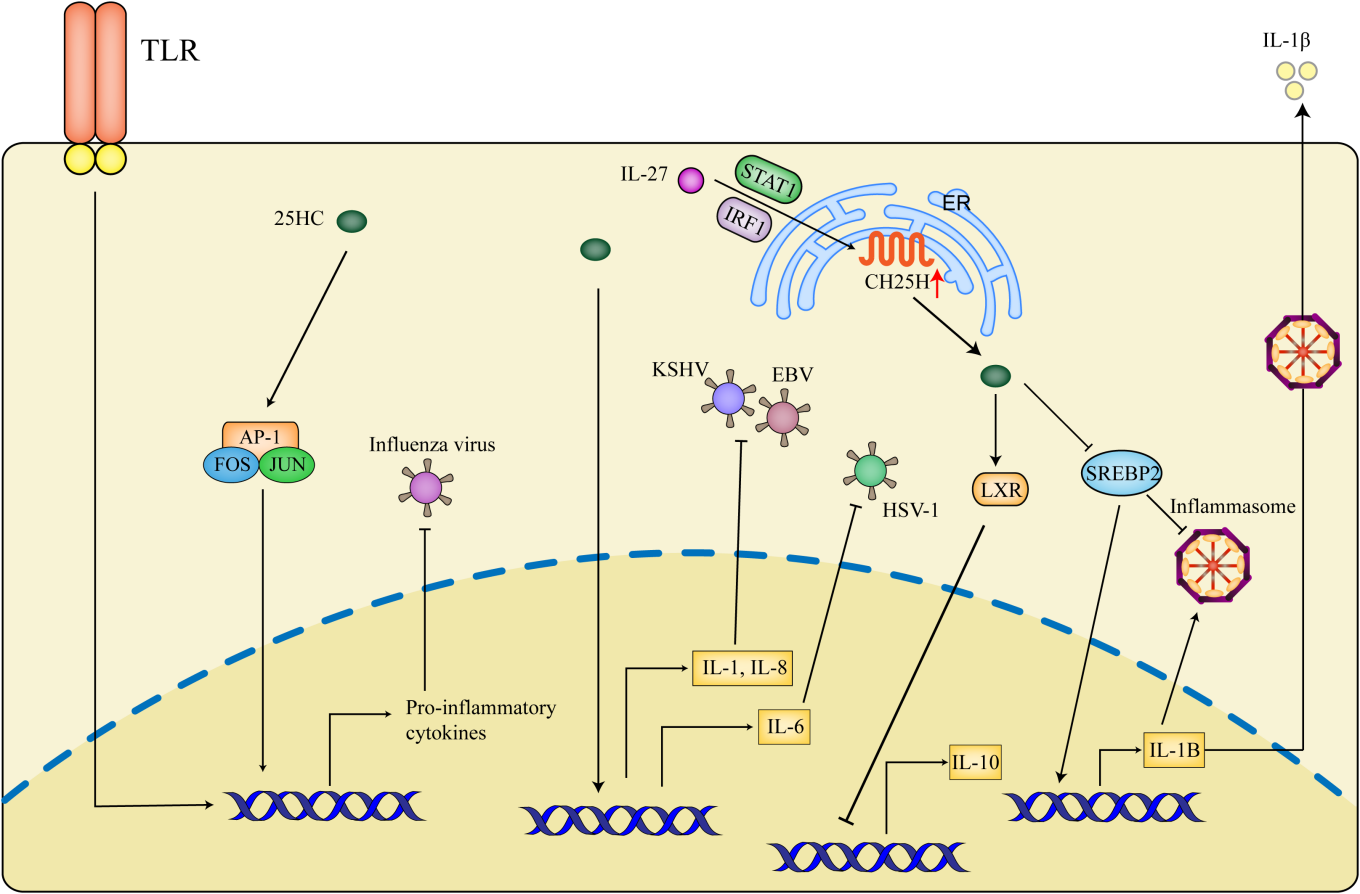
Inflammatory effects of 25HC in antiviral activities. 25HC exerts dual effects on inflammation during antiviral responses. 25HC acts as an amplifier of inflammation by recruiting AP1 to the (Toll-like receptor) TLR-response genes, moreover, 25HC amplifies the expression of inflammatory genes such as IL-1 (interleukin-1), IL-8 and IL-6 to suppress the viral infection. In addition, IL-27 is shown to promote expressions of CH25H and 25HC in a STAT1 (Signal Transducer and Activator of Transcription factor 1)- and IRF1- dependent manner and thus inhibit IL-10 production through LXR. 25HC-induced inhibition of SREBP2 decreases the transcription of IL-1B and secretion of IL-1β and represses the inflammasome activity. The precise condition of 25HC maintaining correct inflammation balance is critical for the control of viral infection, which is not defined nowadays.

In a mouse macrophage model, it is shown that 25HC exerts pro-inflammatory effects partially through mediating the recruitment or retention of AP-1 components at the promoters of TLR-response genes. Interestingly, 25HC inhibits influenza infection *in vitro* while the deletion of CH25H is protective against influenza infection *in vivo* by decreasing the inflammation response (108). 25HC blocks the infection of KSHV (Kaposi’s sarcoma herpesvirus) in primary endothelial cells and the RNA sequencing results show that 25HC can induce inflammatory cytokines such as IL-8 and IL-1α. Besides, IL-1 and IL-8 pathways also are induced by 25HC in primary B cells infected with EBV (Epstein-Barr virus) (109). The proinflammatory effect of 25HC is also demonstrated in HSV-1 infection, the pre-treatment with 25HC enhances IL-6 production and results in augmentation of interleukin’s total secretion, meanwhile, the antiviral potential of IL-6 against HSV-1 *in vitro* is verified (110). Conversely, the anti-inflammatory function of 25HC has also been described. 25HC is shown to inhibit the secretion of IL-1β by suppressing the inflammasomes (111), in the meantime, SREBP2 can activate the NLRP3 inflammasome in the endothelium (57). In addition, 25HC is proven as a potential antiviral agent against ZIKV and reduces inflammation and cell death in ZIKV-challenged U-87 MG glial cells (112). Of note, the content of 25HC and level of CH25H are specifically induced by IL-27 in type 1 regulatory T cells (TR1), furthermore, 25HC is shown to negatively regulate the production of IL-10 via LXR signaling (113). In consideration of the inhibitory effect of 25HC on SREBP2, the anti-inflammatory roles of 25HC mediated by SREBP2 are also identified. 25HC reduces the process of SREBP2 to suppress IL-1B transcription and IL-1-driven inflammasomes (58) (**Figure 4**).

25HC treatment maintains the homeostasis of inflammation, the context dependence of its roles between proinflammation and anti-inflammation, as well as its mechanisms *in vivo* remain uncertain. Determining the *in vivo* range under which 25HC exerts dual effects on inflammation may help to ensure the correct balance of inflammation and to simultaneously control the viral infection by repressing the inflammasome-induced organism damage.

## 7 25HC and transforming growth factor-β

Transforming growth factor-β (TGF-β) signaling plays diverse roles in regulating cellular growth, immune response and oncogenesis. In the past years, multiple studies regarding TGF-β mainly focused on its effect on cancer (114), EMT (Epithelial–mesenchymal transition) (115), lung fibrosis and inflammation, its roles in other diseases were neglected. Recently, its ability to regulate viral infection is identified. The nucleocapsid protein of SARS-CoV is shown to promote TGF-β responses in a Smad-3-specific manner and impede apoptosis in SARS-CoV-infected HPL1 (human peripheral lung epithelial) cells (116). In the severe Covid-19 patients, the researchers analyze the peripheral plasmablasts using single-cell transcriptomes and come up with a result that SARS-CoV-2 induces TGF-β-dominated chronic immune reaction (117). TGF-β also participates in the regulation of the lipogenic process by HCV. HCV genotype 3a induces higher expression of TGF-β and inhibition of PPARα expression and fatty acid oxidation. Besides, HCV-related lipid accumulation is associated with activation of TGF-β (118). Considering the regulatory effect of TGF-β on lipid production, in our previous study, we administrated 25HC to further investigate the role of TGF-β in a PDCoV-infected model *in vitro*. The expression of TGF-β signaling can be dramatically induced by PDCoV infection, which is reversed by 25HC. The application of TGF-β inhibitor reveals its regulatory effect on the genes related to cholesterol metabolism (87). The thorough mechanism of TGF-β in viral infection is yet to be fully demonstrated, but it might be a potential approach for viral invasion and target for 25HC action.

## 8 25HC and posttranslational modification

During the viral invasion, various host machineries are exploited to modify viral proteins, including structural proteins and nonstructural proteins, for survival. One of the critical protein modifications is called glycosylation, which means the addition of glycans to proteins (119). On the one hand, glycosylation of envelope protein is important for the virus to anchor the cytomembrane receptors, on the other hand, glycosylation also helps viruses to decrease the risk of being recognized by the host immune system (120). Lassa virus (LASV) is a member of arenaviridae, its glycoprotein precursor needs to be cleaved into GP1 and GP2, of which the oligomer forms spikes of virions (121). Besides, the cleavage is dominated by glycosylation (122).

The inhibitory effect of 25HC on LASV production is firstly verified in Huh7 cells, after 48 h, the treatment of 25HC increases the GP1 mobility on SDS-PAGE gels and leads to abnormal GP1 production containing more immature N-glycans. Meanwhile, the abnormal GP1 is incorporated into fresh LASV virions and thus impairs their infectivity (123).

In addition to glycosylation, another important post-translational modification named prenylation is also shown to play critical roles in viral life span (124). Whether posttranslational modifications act as antiviral targets of 25HC and the underlying mechanisms are novel angles to uncover the antiviral potential of 25HC.

## 9 Discussion and perspective

In the last years, host cholesterol was identified as a potential antiviral target, in what manner to command cholesterol against viral infection becomes the key point. The repurposing of cholesterol-modifying drugs brings hope to the control of highly pathogenic viruses. Repurposing of antifungal drugs including itraconazole and posaconazole was found to play effective roles in restricting IAV, Ebola virus and SFV (Semliki Forest virus) by regulating cholesterol homeostasis (125–127). Besides, the antidepressant drug fluoxetine targeting acid sphingomyelinase exerts antiviral effects on SARS-CoV-2 and Ebola virus by inducing impaired endosomal acidification and sequestered cholesterol within endosomes (127–129). Statins, the broadly applied cholesterol-lowering drugs also serve as potent antiviral agents against different stages of virus cycle (130). Fenofibrate, a drug decreasing triglyceride and LDL (low-density lipoprotein) cholesterol, restrains the replication of SARS-CoV-2 and alleviates its pathogenesis by regulating lipid metabolism (131).

As a derivate of cholesterol, the negative regulatory role in cholesterol metabolism has already been proved. Besides, it is clear that 25HC has antiviral abilities against diverse viruses. Except for its direct function of stimulating interferon, as highlighted in this review, the last decades have discovered signaling pathways involved in mediating antiviral responses of 25HC. According to the effect of 25HC on cholesterol metabolism, a question has been raised whether specific inhibitors of cholesterol biosynthesis have similar antiviral abilities, such as statins. Given the important influences of lipid droplets on interferon response, what are the exact mechanisms mediating lipid droplets-associated immune response? In consideration of its dual effects on autophagy and inflammation, the details of the mechanisms and how it is integrated with another signaling still need a mass of studies to decipher. Furthermore, it is also unknown whether and how the concentration of 25HC *in vivo* affects its potency. This in turn raises the question of its prospect of clinical antiviral therapy.

Apart from the biological function of 25HC, the structural biochemical characteristic of itself also needs to be concerned. Remarkably, a small chemical group such as a single hydroxyl group can make cholesterol become an antiviral agent and inflammation regulator. Could we utilize chemical approaches to create similar antiviral compounds? Meanwhile, the chemical structure of 25HC results in that it might modify the interaction between proteins and membranes (132), which might function as an antiviral mechanism. Based on this feature, fusion inhibitor peptides containing 25HC are developed to enhance antiviral spectrum and address the resistance (133, 134). In addition to the existing experimental data, some studies on OMIC strategies also predicted the possible mechanism of 25HC. The upregulated protein by 25HC such as nuclear factor-kappa-B p100 subunit (NFKB2) and down-regulated proteins by 25HC such as HMG-CoA reductase and isopentenyl-diphosphate delta-isomerase 1 fit with the previous studies, meanwhile the down-regulated proteins interferon-induced transmembrane protein 3, mannose-6-phosphate receptor and junctional adhesion molecule A pointed novel directions for the likely antiviral mechanisms of 25HC (135).

The complex regulatory effects of 25HC on inflammation and autophagy imply that we should not consider its role as an enhancer or inhibitor simply, but as a dynamic mediator to restore the imbalance of host cells challenged with viral infections. In addition, the regulatory relationship between cholesterol metabolism and other pathways still needs lots of work to decipher. Taken together, we can expect that deeper research into molecular mechanisms and structure modification can help to explain questions about the antiviral effects of 25HC and similar oxysterols. In addition to the individual administration of antiviral drugs, the combination of antiviral drugs and cholesterol-modifying drugs exhibits good therapeutic effects (136), with the advantage of avoiding or impeding the development of resistance. Considering that the prominent antiviral potential of 25HC has been demonstrated in multiple animal experiments, we can also predict further research into this sort of compound that derives from host metabolism can serve as a drug candidate to contain emerging infectious pathogens alone or in combination.

## Author contributions

JZ: Writing – original draft, Writing – review & editing. YZ: Writing – original draft. XW: Writing – review & editing. JW: Funding acquisition, Supervision, Validation, Writing – review & editing.
